# Upstroke wing clapping in bats and bat-inspired robots offers efficient lift generation

**DOI:** 10.1098/rsif.2024.0590

**Published:** 2025-02-19

**Authors:** Xiaozhou Fan, Alberto Bortoni, Siyang Hao, Sharon Swartz, Kenneth Breuer

**Affiliations:** ^1^ Center of Fluid Mechanics, School of Engineering, Brown University, Providence, RI, USA; ^2^ Department of Ecology, Evolution, and Organismal Biology, Brown University, Providence, RI, USA

**Keywords:** jet propulsion, bio-inspired robotics, animal flight, reduced-order modelling, experimental fluid dynamics, unsteady aerodynamics

## Abstract

Wing articulation is critical for the efficient flight of bird- and bat-sized animals. Inspired by the flight of *Cynopterus brachyotis*, the lesser short-nosed fruit bat, we built a two-degree-of-freedom flapping wing platform with variable wing folding capability. In the late upstroke, the wings ‘clap’ and produce an air jet that significantly increases lift production, with a positive peak matched to that produced in the downstroke. Though ventral clapping has been observed in avian flight, the potential aerodynamic benefit of this behaviour is yet to be rigorously assessed. We used multiple approaches—quasi-steady modelling, direct force/power measurement and particle image velocimetry (PIV) experiments in a wind tunnel—to understand critical aspects of lift and power variation in relation to wing folding magnitude over Strouhal numbers at *St* = 0.2–0.4. While lift increases monotonically with folding amplitude in that range, power economy (ratio of lift/power) is more nuanced. At *St* = 0.2–0.3, it increases with wing folding amplitude monotonically. At *St* = 0.3–0.4, it features two maxima—one at medium folding amplitude (approx. 30°) and the other at maximum folding. These findings illuminate two strategies available to flapping wing animals and robots—symmetry-breaking lift augmentation and appendage-based jet propulsion.

## 1. Introduction

**Figure 1.**
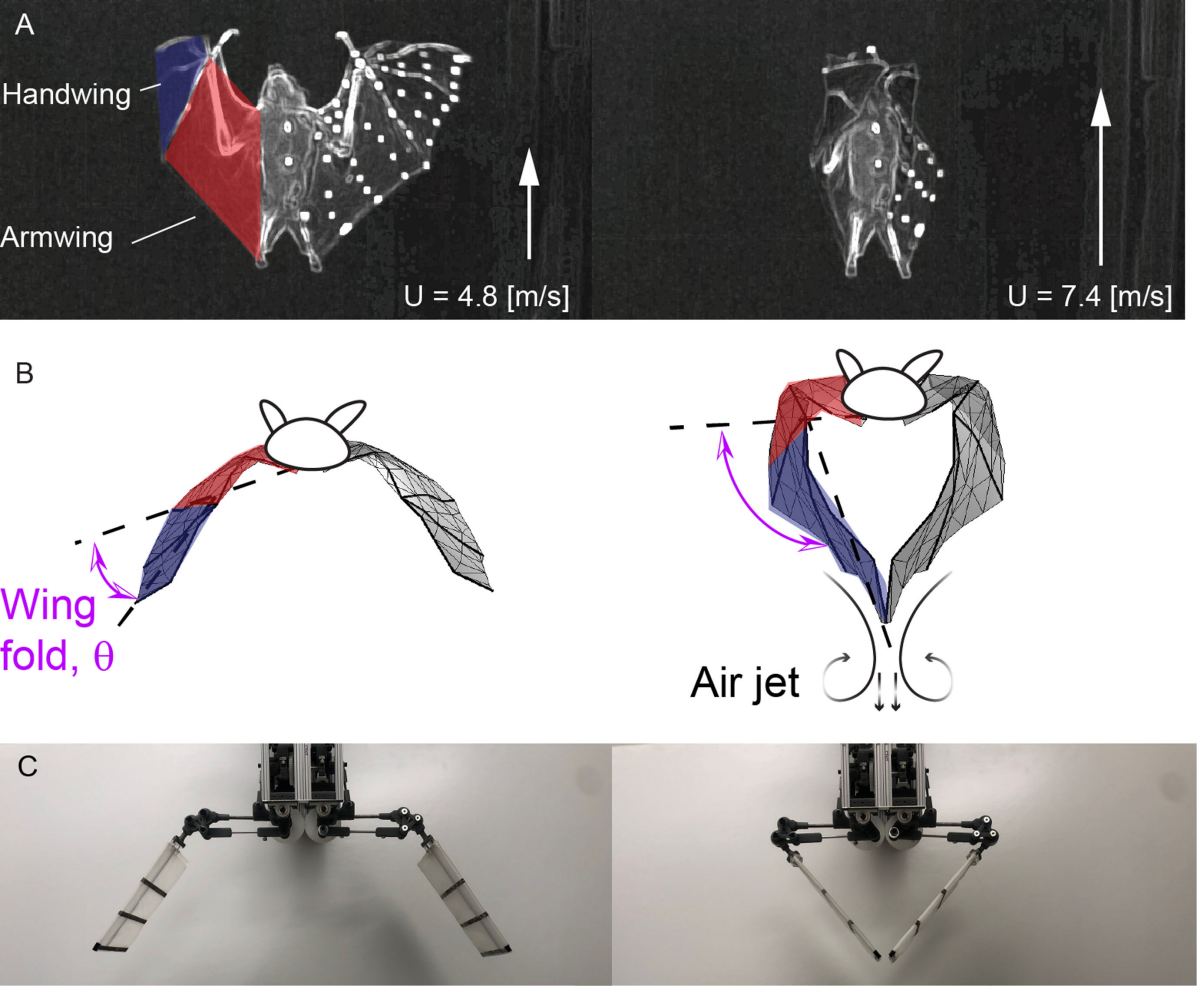
(A) Ventral or inferior view of *Cynopterus brachyotis* in straight and level flight at the start of upstroke (Riskin *et al*. [6] and videos or raw dataset provided by Fan *et al*. [5]) at two different speeds. White markers on the wings are used for motion tracking. The wing folding angle $\theta_{o}$ at U=7.4 m s^−1^ is clearly larger than that at U=4.8 m s^−1^, where the two handwings clap together as a result of large wing folding angles at higher flight speeds. (B) Frontal views of the meshed bat wings based on motion tracking and stereo-triangulation [5,6]. For a flight speed of U=4.8 m s^−1^, at the moment of the maximum wing folding, the wingtips remain widely spaced, but for U=7.4 m s^−1^, the two wingtips approach and clap, producing a downward-directed air jet, which generates additional lift. The dashed lines here represent the angle between armwing and handwing as defined in [4]. (C) Photo of the two-degree-of-freedom, bat-inspired robot, ‘Flapperoo’, capable of performing independent flapping and folding motion.

Bats fly with highly articulated wings; in particular, the kinematics of the handwing, from the wrist to the wingtip, vary significantly with flight speed [1–5]. During upstroke, in addition to wing elevation, bats’ wrists flex, which rotate the handwing with respect to the armwing about a chord-wise axis through the wrist and fold the wings [3] (figure 1A). It has been suggested that folding reduces the inertial power of wing elevation [7] and that the reduced wing wetted surface area leads to a decrease in negative lift [2,3]. During the late downstroke, due to wing folding, the additional angular velocity of the handwing increases its effective angle of attack and effective velocity, which contribute to greater lift [8,9]. It is therefore no surprise that bio-inspired robotic flyers with wing folding capability also demonstrate superior performance, such as flight endurance [10,11], compared with those that flap but do not fold [12].^1^

In some cases, bats fold their wings so much during upstroke [6] that the two wingtips touch and clap (figure 1B). This phenomenon occurs not only in multiple bat species [17,18] but also in small birds that hover, such as warbling white-eyes (*Zosterops japonicus*) and Gouldian finches (*Erythrura gouldiae*) [19,20].

In this article, we report on efforts to understand how wing folding may influence flight performance in bats in terms of aerodynamics and energetics. This is difficult or impossible to ascertain in live animals and thus we designed, built and tested a mechanical robot, ‘Flapperoo’, that abstracts key features of the bats’ wingbeat kinematics, allowing us to manipulate relevant variables.

By combining direct force/power measurements, time-resolved particle image velocimetry (PIV) and quasi-steady modelling, we explain the measured forces/power using validated blade-element momentum theories using the observed kinematics [4,5]. Additionally, the measured forces, power and PIV help to determine the effectiveness and limitations of the reduced-order computational model. If folding enhances the flight performance of membrane wings, an exploration of this enhancement using a robotic platform is a crucial first step in the design process, which identifies how best to incorporate wing folding into a flapping wing robot.

However, there are as yet no systematic studies on the unique wing folding/clapping phenomenon. Few robotic studies have implemented the actively actuated wing folding motion observed in bat and bird species [10,21] and, to the best of our knowledge, none have reported the power and aerodynamics of varying wing fold amplitudes. Both Chen and Yeh [21] and Kashi *et al*. [22] built robotic flappers to replicate folding in wings or during flipper clapping, but no lift benefits were observed.

**Figure 2.**
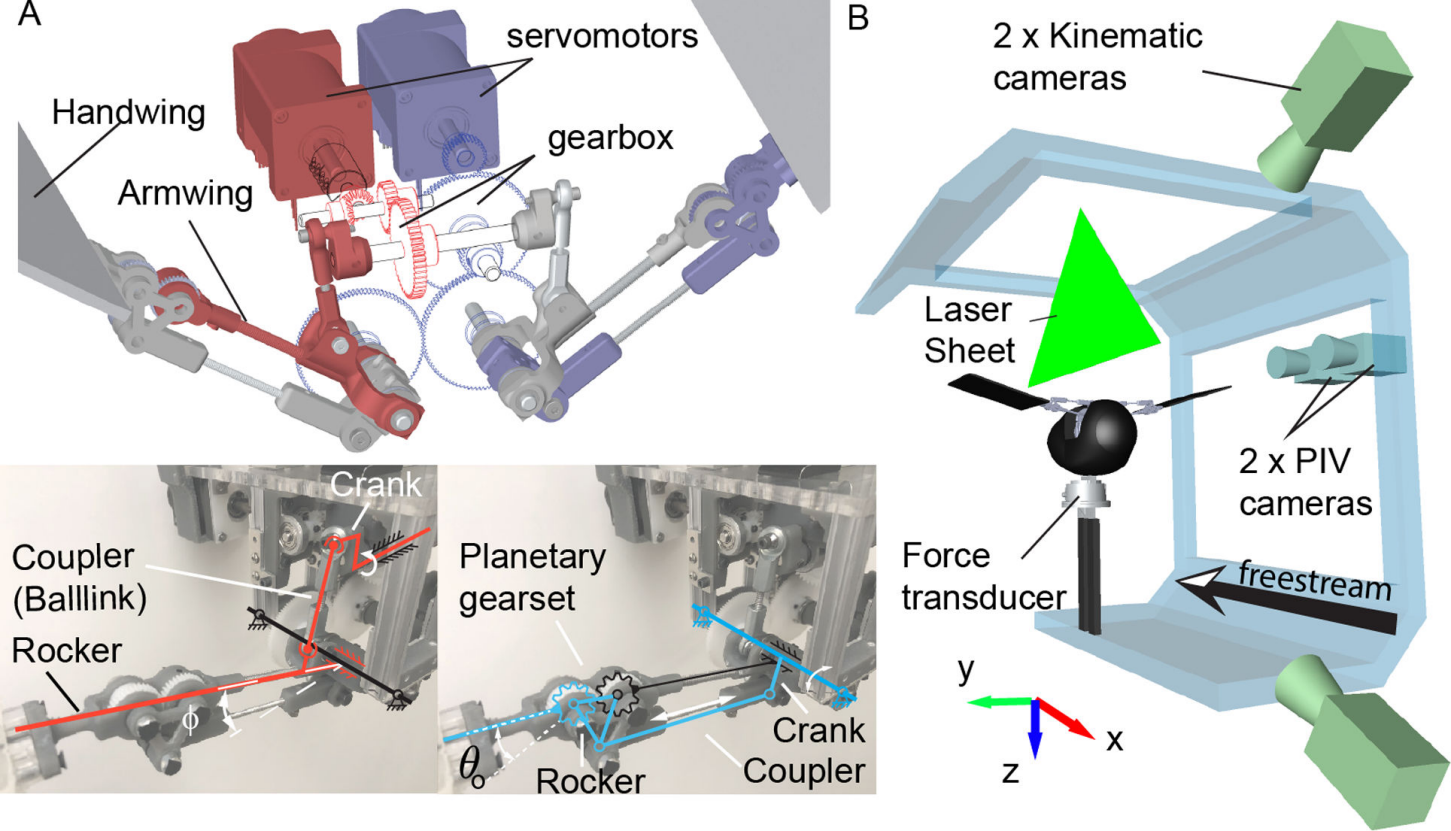
Model design and experimental set-up. (A) Flapperoo performs independent wing flapping (mechanism in red, with fixed magnitude ϕ) and folding (mechanism in blue, with magnitude denoted as $\theta_{o}$) motions, which are realized by two four-bar linkages, each driven by a servo motor. The left and right-wing motions are symmetric. (B) Experimental set-up. Flapperoo is mounted on a six-axis force/torque transducer in the wind tunnel. The freestream points to the negative of the x-axis and the z-axis is the direction of gravity. The model is mounted upside down (ventral side up) for unobstructed access to the PIV laser sheet.

To assess the role of wing folding in flapping flight, both flapping and folding motions are programmable for Flapperoo. The bio-inspired robot allows direct measurement of force and power in a manner that is not possible from animals. The wing movement mechanism is composed of two four-bar linkages, driven by two servo motors, in which one controls wing flapping, a movement of the ‘armwing’, and the other controls wing folding, a movement of the ‘handwing’ relative to the armwing (figures 1C and 2A, and refer to §2 for a more detailed description).

We define up- and down-strokes by the motions of the armwing and set them to be equal in duration. Studies of hovering Japanese white-eyes, *Zosterops japonicus* and Gouldian finches, *Erythrura gouldiae*, have classified wing clapping as part of the downstroke [19,20], based on the trajectories of the wingtips. However, the primary flight motor of vertebrates is the pectoralis major muscle [23], and for this reason, we deem the motion of armwing to be a more accurate reflection of up- versus down-stroke. Additionally, the cyclic motion of the armwing is more stable and consistent than that of the deforming handwing. Using this framework, bat wing clapping occurs after the armwing has reached the bottom of the downstroke and begun the upstroke, although the handwings, which perform the clap, are still moving downwards (ventrally) at this point in the wingbeat cycle. For all the wing folding cases we considered, folding begins in the late downstroke.

We carried out tests over a range of freestream velocities and wing folding amplitudes in a wind tunnel (figure 2B). This corresponded to Strouhal numbers, St (defined as St=fA/U, where f is the flapping frequency, A is the vertical wingtip displacement when there is no folding and U is the freestream velocity [6,24]) over the range of 0.2–0.4—a particularly relevant range for flying animals [25]. We used the measured robot kinematics as input to a quasi-steady computational model [4,5,9], which estimated aerodynamic force and power. Then, we compared force and power, estimated from the computational simulations, with the directly measured forces (from a force transducer) and power consumption (from the motor’s driving current and voltage). To quantitatively characterize the air jet produced, we measured the flow in a vertical (parasagittal) plane between the two wings (figure 2B) using time-resolved PIV and performed a Reynolds-averaged control volume analysis to assess the forces associated with this flow [26,27].

## 2. Material and methods

**Figure 3.**
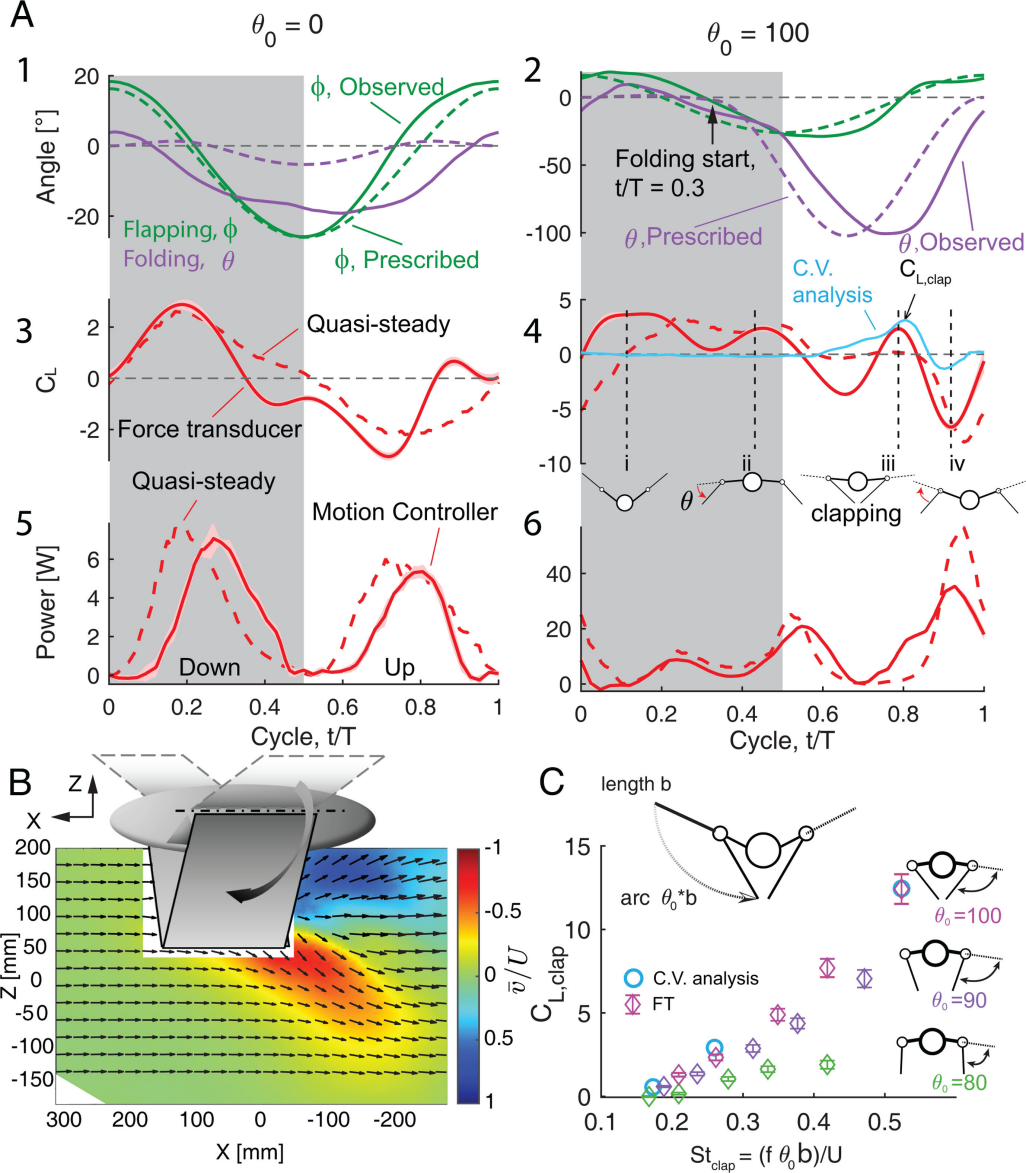
(A) Kinematics, lift coefficient and power consumption over the wingbeat cycle for the near-zero ($\theta_{o}=0$) and maximum folding angle cases ($\theta_{o}=100^{\circ }$, wings clap); freestream velocity, U=4 m s^−1^. Solid line: observed left-wing kinematics; dashed line: commanded motion; left and right-wing movements are symmetric. Grey shading indicates downstroke as defined by armwing motion. Force coefficients of lift and power measurements: solid lines indicate average force (40 cycles) and shading indicates RMS variance. $C_{L,clap}$ is defined as the local peak of the $C_{L}$ during wing clapping. The control volume analysis, derived from PIV, is presented as a solid blue line. Four snapshots (*i, ii, iii* and *iv*) highlight instances of interest in the wingbeat cycle (figure 4). (B) Phase-averaged (40 cycles) PIV velocity field at time point (*iii)*, maximum wing folding during clapping. The instantaneous velocity field is depicted by black arrows, with vertical components normalized by freestream velocity (U=4 m s^−1^). (C) Scaling of $C_{L,clap}$ as a function of a locally defined Strouhal number, $St_{clap}$, from transducer-measured forces (FT) for three folding angles and from the PIV-derived control analysis calculations. The uncertainty bars are the standard deviation calculated from 40 cycles.

We designed and built a two-degree-of-freedom (d.f.) flapping-wing robot, Flapperoo, capable of both wing flapping and folding. The flapping motion (red, figure 2A) is realized by a four-bar linkage mechanism. A crank rotates continuously in one direction to pull/push the ball link (coupler), causing the armwing (rocker) to oscillate up and down; the angle between the rocker and the horizontal plane is denoted as the flapping angle ϕ, which is fixed. To fold the handwing (blue, figure 2A), an independent crank is driven reciprocally, dragging the coupler to move in and out. This motion pivots the spur gear (planet gear, blue) on the rocker (carrier) to rotate against the fixed gear mounted on the armwing (sun gear, black), so that the folding angle is amplified (2:1 ratio). The handwing is fixed to the spur gear and the angle it makes relative to the armwing (black) is the wing folding angle, denoted as θ. The folding of wings begins at t/T=1/3 of a cycle, in the downstroke (black arrow in figure 3A(2)) and is sinusoidal. A streamlined body for Flapperoo was hand-sculpted using foam and houses the flapping mechanism (figure 2B). The body and wings were painted matt black to reduce light scattering during PIV experiments.

**Figure 4.**
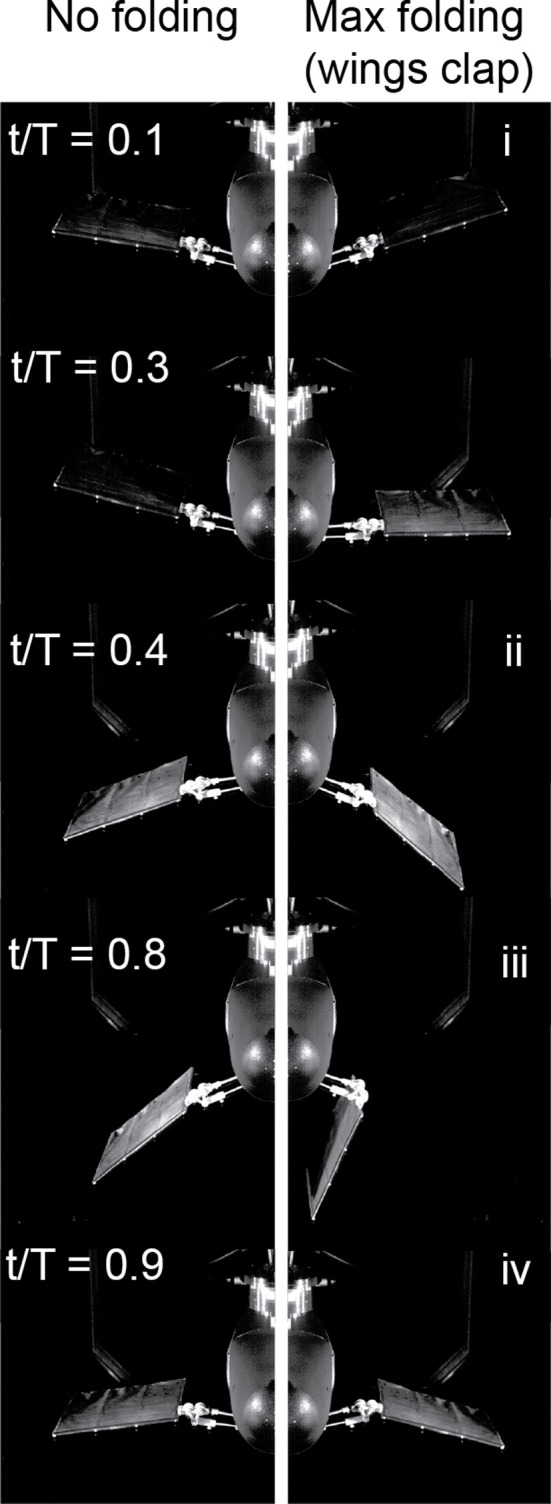
Frontal view of wing kinematics in the wind tunnel at five key snapshots of one wingbeat cycle. Left and right panels are for cases without folding ($\theta_{o}=0$) and maximum folding ($\theta_{o}=100^{\circ }$, wings clap), respectively.

The wing ribs were laser-cut from flat balsa wood strips with a chord length of c=200 mm and a thickness of 5 mm. The equally spaced ribs were glued onto a 200 mm carbon-rod spar before being covered with ripstop, kite-cloth fabric. The handwing and armwing are equal in length. The wing assembly weighs 10 g. To isolate the effect of handwing structure, the 100 mm armwing skeletal frame is not covered with fabric.

The flapping and folding movements are actuated by two brushless rotary servo motors with integrated encoders (BE163CJ-NFON, Parker Hannifin Corp., Rohnert Park, CA), controlled by a servo controller (DMC-4060, Galil Motion Control, Rocklin, CA). The motors are controlled using custom-written code in software developed for the controller (GDK, Galil Motion Control) and operate in ‘PVT’ mode, where the user defines a list of target positions and velocities at a series of defined times. The controller then moves the motor through a profile that reaches each target position at the target time, moving at the target velocity. The list of target values was calculated using a MATLAB script.

The flapping motor was programmed to execute a constant-speed rotation. We used a testing frequency of 3 Hz for all investigations reported here. The folding motor was programmed to start the fold motion at t/T=1/3 and to end at t/T=1. Due to the coaxial nature of the rocker during flapping and the crank during folding, friction will cause one to rotate with the other and thus, even for the no-folding case ($\theta_{o}=0$), we drive folding at a low level to counter this friction at a non-zero amplitude (figure 3A(1)). This small base offset is added to all other cases.

The robot was tested in a closed-loop wind tunnel at Brown University [28] (figure 2B). The test section is 1.2×1.2 m in cross-section and 4 m long. We varied the freestream velocity to achieve the desired Strouhal number, St=fA/U, where A is the vertical wingtip displacement when there is no folding ($\theta_{o}=0$) and U is the freestream velocity for the fixed 3 Hz testing frequency [6,24]. We introduce an additional, local Strouhal number to describe jet scaling during wing clapping, $St_{clap}=f\theta_{o}b/U$, where b is the length of each individual handwing.

The robot was mounted on a six-axis force/torque sensor (Gamma IP65, ATI Industrial Automation, NC) and force/torque measurements were recorded using an A/D converter (USB-6343, National Instrument, TX) at 1000 Hz. Angular velocity and torque were recorded at 512 Hz from the motors using the Galil motion controller and their inner product was computed to yield mechanical power. The net lift was normalized by the dynamic pressure of the freestream and the wing area: $1/2\rho U^{2}2*bc$. The net power was similarly normalized by the dynamic pressure of the freestream and the wing area and chord as $1/2\rho U^{3}2*bc$.

For each trial, we first acquired data with the handwings attached and then removed the handwings and repeated each measurement with the same set of parameters. The trials without handwings record the inertial forces and torques associated with the motion of the mechanical components (e.g. gears, cranks, etc.). Note that the inertia power from handwing (approx. 9[g]) alone is negligible and is thus not considered. A Butterworth low-pass filter was applied to these data with a cut-off frequency $f_{c}=5f\;(15\;Hz)$. The net aerodynamic force or power is the difference between these two conditions (Bahlman *et al*. [14]).

White reflective markers, placed at the leading edge of the wings, were tracked from video acquired by two Phantom Miro 340 cameras (Vision Research Inc.) at 800 fps. The video and force data acquisition were synchronized using an Arduino microcontroller. The cameras were calibrated and the video was digitized using DLTdv8 [29]. Subsequently, wing kinematics were used in a quasi-steady flapping flight model to predict aerodynamic force and power [4,5,9,13].

The PIV experiment used a Nd:YLF double-pulsed laser (DM30, Photonics Industries, Ronkonkoma, NY), employed at 500 Hz with an energy output of approximately 30 mJ/pulse. The vertical laser sheet was aligned with the flow direction and passed through the Flapperoo’s body midline, the centre of the gap between the wingtips (figure 2B). The test section was seeded with neutrally buoyant, helium-filled soap bubbles (diameter 0.3 mm), released upstream of Flapperoo (Lavision Inc., Germany). The bubbles illuminated by the laser sheet were imaged by two high-speed cameras (Photron NOVA R2, 2048 × 2048 pixels), positioned side by side with overlapping fields of view, producing a combined field of view of 600×400 mm. We used DaVis PIV software v. 10 (LaVision Inc., Germany) to perform image cross-correlation and MATLAB code to obtain time-resolved phase-averaged velocity fields in x and z (N=83 bins/cycles), where each bin contained 40 repetitions [30].

The aerodynamic forces that result from wing clapping, $\vec{L}$, were derived from the Navier–Stokes equation using the control volume (CV) analysis approach (figure 3C) (please refer to supplementary materials for the detailed derivation, also see Bohl & Koochesfahani [26] for a similar but simpler analysis),


(2.1) $$\begin{array}{r} \vec{L}=\underset{\text{Acceleration}}{\underbrace{\frac{\partial }{\partial t}\int_{C.V.}\rho \vec{v}dV}}+\underset{\text{Momentum flux}}{\underbrace{\int_{\text{C.S. }}\rho \vec{v}(\vec{v}\cdot \vec{n})dA}}-\underset{\text{Pressure}}{\underbrace{\int_{\text{C.S. }}-p\cdot \vec{n}dA}}, \end{array}$$

where ρ is the air density, $\vec{v}$ the velocity field of the flow, $\vec{n}$ the surface normal of each control surface (C.S.) and p the pressure acting on each surface.

All derivatives were evaluated using a Savitzky–Golay filter (order 2, frame length 5) [31]. The first term is a volume integral of the acceleration field along the vertical *z*-direction, the second term is the transport of *z*-axis momentum across each downstream boundary surface of the control volume and the third term is a line integral that gives the pressure difference between the bottom and top surfaces in figure 3B (Surface 3 and 4 in supplementary materials figure S10).

To obtain the clapping lift coefficient $C_{L,clap}$, we assume that the width of the jet is uniform into the depth of the measurement plane (y-axis in figure 2B) around the time of wing clapping and that the width is of the order of twice the length of the handwing, 2b, consistent with PIV studies of wing clapping in birds [19,20]. Normalized by the dynamic pressure, q and surface area of the whole wing, S=2bc, the lift coefficient from the PIV experiment is thus $C_{L}=L2b/qS=L2b/q2bc=L/qc$.

## 3. Results

### 3.1. Robot performance

We present results for two cases at the extreme of the robot’s kinematic space: near-zero wing folding, $\theta =0^{\circ }$ and extreme wing folding with a wing clap $\theta =100^{\circ }$ (figure 4). For both cases, the freestream U=4 m s^−1^, which corresponds to St=0.21.

We observed a smooth flapping motion that followed the prescribed motion with reasonable fidelity (figure 3A(1) and A(2), green lines). For the near-zero prescribed folding angle case (figure 3A(i)), the folding angle of the handwing varies passively over the wingbeat cycle, with values ranging from $-20^{\circ }$ to $5^{\circ }$. The observed folding angle is closer to the prescribed value for the larger folding angle case, with values similar to those prescribed in magnitude but with a phase lag of about t/T=0.1 (figure 3A(2)). See also the total angles of the handwing with respect to the mid-stroke plane in electronic supplementary material, figure S1.

### 3.2. Lift

The mechanics and energetics of the two folding cases differ substantially. The magnitude and trend of the aerodynamic lift coefficient, $C_{L}$ ($C_{L}=F_{z}/0.5\rho U^{2}S$, where $F_{z}$ is the dimensional force, ρ is the fluid density and S is the total area of the two handwings), for the case of near-zero folding are similar when measured by the force transducer (FT) and predicted by our quasi-steady model [4,5] (figure 3A(3)). Positive lift is generated during the downstroke and negative lift during the upstroke. The quasi-steady model diverges from the FT data primarily by an overprediction (more positive lift) of $C_{L}$ in late downstroke/early upstroke (t/T= 0.3–0.6) and an underprediction (less positive lift) around the upstroke to downstroke reversal (t/T= 0.8–1). For the extreme wing folding case (figure 3A(4)), downstroke is responsible for most of the positive lift (t/T= 0–0.5), but there is more variation across the wingbeat cycle and a more pronounced difference between the lift estimates of the measurement and the quasi-steady model. Specifically, the model produces a more pronounced underprediction of $C_{L}$ in comparison with the FT measurement at the beginning of the downstroke (snapshot i) and slightly overpredicts lift around the mid-downstroke (between snapshots i and ii). The quasi-steady and FT measurements agree well late in the downstroke (snapshot ii). At time t=0.8, when the wing claps (figure 3A(4), snapshot iii), the quasi-steady model fails to predict the peak in lift generation $C_{L,clap}$ in figure 3A(4). Towards the end of the upstroke (t/T∼0.9, figure 3A(4), snapshot iv), when the wing has completed the upstroke movement from the ventral to the dorsal side of the body and prepares for the next downstroke, the quasi-steady model captures the negative lift measured by the force transducer, albeit with a slight overprediction and phase delay.

### 3.3. Power consumption

Computations of the quasi-steady model predict moderate peaks in power consumption during the middle of the downstroke and the upstroke (figure 3A(5)). These predictions are supported by the power measurements from the robot, although there is a delay (with the quasi-steady model leading by t/T=0.1) in the power maximum. When wing folding is at its maximum ($\theta_{o}=100$, figure 3A(6)), the model predicts a more complex time course for power consumption, but the overall pattern of power use measured from Flapperoo is a close match to the simulation, with the exception of an underprediction of power at (snapshot iii) (t/T∼0.8) and a sharp rise in predicted power late in the upstroke at t/T∼0.9, (snapshot iv), which is not observed by the measurement.

### 3.4. Velocity field

PIV measurements of the flow field along the midline plane of Flapperoo show a prominent jet that is directed downwards and to the rear of the ‘animal’, which appears as a result of wing clapping at t/T∼0.8 (snapshot iii and figure 3B). Because the velocity measurement is restricted to the centreline plane between the two wings, the CV analysis of the aerodynamic forces associated with this jet (figure 3A(4); blue line) is only expected to yield accurate force estimates during the portion of the wingbeat cycle in which wing clapping occurs (refer to §2 for a more detailed description). During this time period, $C_{L}$ agrees well with FT measurement. During other portions of the wingbeat cycle, flow in this plane closely follows the freestream and $C_{L}$= 0 (see electronic supplementary material, video S1). The $C_{L}$ calculated from the CV approach is independent of the CV boundary (once a large enough volume is chosen).

The jet is produced as a result of the left and right wings clapping, ‘squeezing’ air out. We predict that the strength of the jet created by two wings clapping will depend on the distance between the wingtips, which, in turn, depends on the clapping amplitude, $\theta_{o}$ and the speed of the approaching wings, $f\theta_{o}b$, where f is the flapping frequency and b is the length of the handwing, measured from the wrist to the wingtip. We define a local lift coefficient, $C_{L,clap}$, from the peak lift force during the clap (figure 3A(4)), and observe a clear dependence of $C_{L,clap}$ on the clapping amplitude, $\theta_{o}$, and the wingtip velocity (normalized by the flight speed to form a clapping Strouhal number: $St_{clap}=f\theta_{o}b/U$) (figure 3*C*).

**Figure 5.**
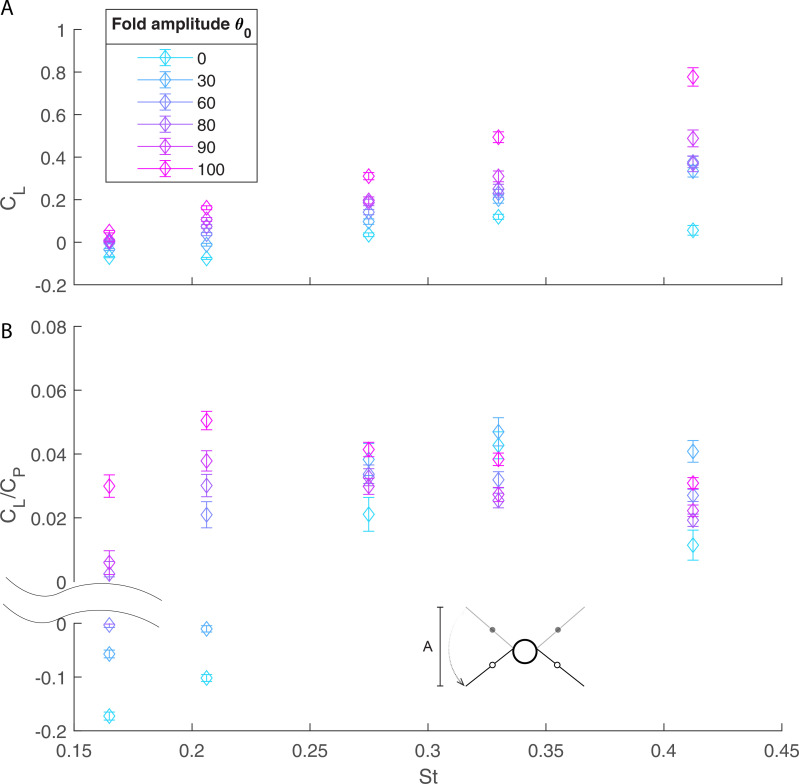
Cycle-averaged (A) lift coefficient and (B) power economy as a function of Strouhal number and maximum folding angle $\theta_{o}$. The aerodynamics of the body and the inertia of the mechanism in motion are subtracted.

Wing folding confers aerodynamic benefits but requires actuation and so incurs energetic costs. Examination of the cycle-averaged lift coefficient, $C_{L}$, and power economy (the ratio of $C_{L}$ to $C_{P}$) as a function of Strouhal number, St, (figure 5A) shows that for the non-folding case, $\theta_{o}=0$, the cycle-averaged $C_{L}$ is close to zero for all values of St (the upstroke–downstroke wing motion is symmetric when wing folding amplitude, $\theta_{o}$, is non-zero and hence no net lift is expected). With wing folding, the cycle-averaged lift coefficient increases with St, up to a maximum value of $C_{L}∼0.8$ for $(St,\theta_{o})=\left(0.41,100^{\circ }\right)$. For a given Strouhal number the lift coefficient increases with wing folding amplitude, $\theta_{o}$, and increases more steeply close to the clapping condition (see electronic supplementary material, figure S2, for $\theta_{o}=90$ to 100).

**Figure 6.**
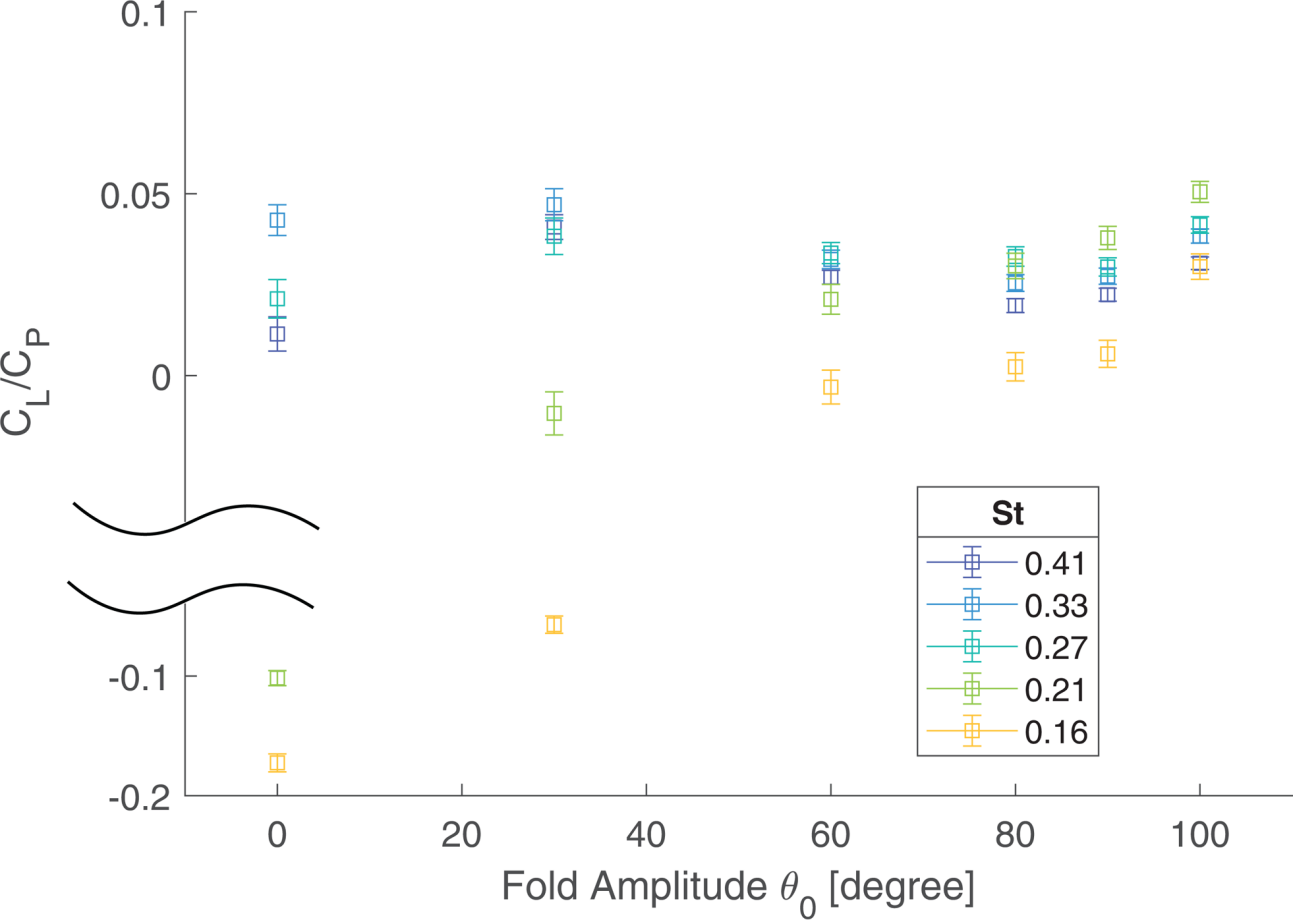
Cycle-averaged power economy as a function of folding amplitudes $\theta_{o}$.

Power economy, $C_{L}/C_{P}$, does not monotonically increase with Strouhal number nor fold amplitude (figures 5*B* and 6). For low to moderate St (St= 0.16–0.27—moderate to fast flight), power economy increases monotonically with folding amplitude $\theta_{o}$ and peaks at the highest fold angle $\theta_{o}=100$. For higher St (St= 0.33–0.41; lower flight speed), the most economical solution to gain lift occurs around $\theta_{o}=30$, although power economy does start to increase again due to wing clapping at high folding amplitude $\theta_{o}$ (figure 6). Thus, at high St, power economy features twin peaks, one at moderate folding and the other at extreme folding amplitude. Note that right around St=0.27, both the folding angle of $\theta_{o}=30$ and 100 are indistinguishably optimal.

## 4. Discussion

The generation of propulsion by jetting for locomotion is well known in the biological world. Jellyfish contract circular muscle fibres in their hemispherical, bell-shaped bodies, shrinking total volume and ejecting water to effect the jet [32]. Circular muscles around the squid funnel of the squid mantle cavity control intake and propulsive outflow of water [33]. The vortex dynamics of these volume reduction-based approaches to jet propulsion has received considerable attention in the fluid dynamics community. Similarly, appendages can generate jet propulsion. Sea lions clap their foreflippers during swimming [34] and wing clapping has been observed in small birds during hovering [19] and by some bats during fast flight [5,6].

In addition to *Cynopterus brachyotis*, the inspiration for this study, biologists have long observed wing-clapping behaviour in bat species from the family Pteropodidae: *Eonycteris spelaea, Macroglossus sobrinus, Eidolon helvum* and species in the genus *Rousettus* [17,18]. The foci of these reports, however, were the clicking sounds generated by wing clapping as a form of biosonar [17,18] and not any potential aerodynamic effects.

A robotic platform that mimics sea lion flippers demonstrated clapping-based jet propulsion but did not show force augmentation associated with the jet [22]. In contrast, we find that lift is significantly enhanced by wing clapping, with a force production close to that generated during the downstroke (snapshot iii in figures 3A(4) and 4). This enhancement comes primarily during the upstroke, not generally considered a lift-generating phase of the wingbeat cycle [2,35,36].

### 4.1. Force estimates from particle image velocimetry

To calculate the lift generated by wing clapping by hovering birds, Chang *et al*. [19,20] conducted frontal and parasagittal plane PIV measurements during the hovering flight of these birds, and a coherent vortex ring is visible as a result of ventral wing clapping. Lift is estimated from the circulation of this vortex ring during the downstroke, under the assumption of an aerodynamically passive upstroke and explains approximately 80% of weight support. Here, we do not assume a vortex ring structure but apply a CV approach, which lends itself well to the spatially and temporally complex flow in terms of pressure, momentum flux and acceleration of fluid by the wing motion.

By phase-averaging the flow field over 40 wingbeat cycles, we found that the contribution from turbulent velocity fluctuations is negligible in estimating $C_{L,clap}$ compared with the mean (phase-averaged) values, suggesting that a coherent vortex ring dominates lift production. This agrees with the observation by Chang *et al*. [19,20].

Net pressure and fluid acceleration start to build well before the wings clap (electronic supplementary material, figure S3), suggesting that the wing–wing interaction occurs much earlier than the actual instant of clapping (see also [37]) and lasts a substantial fraction of a wingbeat (t/T= 0.65–0.8 in electronic supplementary material, figure S3). Indeed, the wings do not need to be physically touching to effect the jet but instead can be separated by a small distance (figure 3C).

### 4.2. Effectiveness and limitations of the quasi-steady model

For the case without wing folding (near-zero case), the agreement between the force measurements and the quasi-steady predictions is generally very good, both in terms of the overall trend and the magnitude of the lift force (figure 3A(3)). Because the wing motion—up- and down-stroke—is symmetric (figure 3A(1)), the model predicts the upstroke and downstroke forces to be equal and opposite and the measurements largely support this—with the deviation due to slight asymmetry in the realized kinematics. Measured and predicted forces differ just ahead of the down- and up-stroke reversals, at t=0.4 and t=0.9, respectively. At mid-downstroke and upstroke (t/T∼0.25 and ∼0.75), the velocity and effective angle of attack (eAoA) peak (electronic supplementary material, figure S4A). As a result, the lift force peaks at these points in the cycle. The wing slows as it reaches stroke reversal and the lift force predicted by the quasi-steady analysis approaches zero. However, the measured lift drops to zero somewhat earlier in the wingbeat cycle.

This discrepancy between measured and predicted lift exposes one of the key weaknesses of the quasi-steady approach: it fails to account for the wake capture around the up- to down-stroke reversal. At the end of the downstroke, a leading-edge vortex (LEV) forms on the dorsal side of the wing [38–40]. This vortex contributes additional lift and, as long as it stays attached, can be modelled accurately by the quasi-steady approach using a generalized lift coefficient [41]. However, for large effective angles of attack, the vortex separates, and when it moves away from the wing, the lift force drops precipitously. Simulations of vortex formation and shedding of a two-dimensional aerofoil have shown that for eAoA = 72°, the vortex is much stronger and sheds more quickly than for the case of eAoA = 4.5°, in which the core is weaker and remains attached [42]. Here, the eAoA at the 3/4-span section for the zero folding case ($\theta_{o}=0$) varies between −40° and 40° (electronic supplementary material, figure S4A). We suggest that at these points in the wingbeat cycle, the LEV is probably shed into the wake, leading to the early drop in the lift coefficient, which is not captured by the low-order model.

For the case of 100° wing folding, the amplitudes of the forces are accurately captured and the overall trends match well, but we do see significant differences between measurements and the model predictions (figure 3A(4)). In particular, at the start of the downstroke (t/T∼0.1), at the beginning of the upstroke (t/T∼0.6) and lastly, mid-upstroke (t/T∼0.8). The first two of these points in the cycle can be explained using the same reasoning as with the straight wing. In this case, the cycle of the effective angle of attack of the wing and its return to zero are delayed due to the kinematics of wing folding (electronic supplementary material, figure S4B) and the eAoA is high (approx. $\pm 50^{\circ }$) at the stroke reversals (t/T=0,0.5) and does not drop to zero until t=0.1 and 0.7, respectively. As before, the quasi-steady analysis predicts zero lift when the eAoA is zero (as it should), while the measurements show that the lift has already dropped to zero ahead of the zero eAoA point due to the LEV separation.

At t/T∼0.65, during the upstroke of the high folding case, $\theta_{o}=100$, the wings are almost perpendicular to the ground (figure 4), leading to a near-zero prediction of $C_{L}$ from the quasi-steady model. The direct force measurement demonstrates a negative trough at this time, which may be due to the LEV on the dorsal side of the wing that generates unfavourable (negative) lift as it separates into the wake. However, shortly after this, at t/T∼0.8 (stage iii in figure 4), while the model still predicts negligible lift due to the vertical orientation of the wing surfaces (figure 4), the force measurements record a positive lift peak, also captured by the PIV measurements. This lift augmentation arises from wing clapping (figure 3B), as a brief jet of air is squeezed downwards, similar to jetting in swimming jellyfish or squid [32]. This momentary rise of $C_{L}$ due to wing clapping is not captured by the quasi-steady analysis, which cannot incorporate wing–wing interactions. The wing motors must provide extra energy to produce the jet, and this additional power requirement is also absent from the quasi-steady model (figure 3A(6), t/T∼0.8).

### 4.3. Aerodynamic force, power and efficiency of wing folding

Birds and bats adjust wing folding amplitude with flight speed [3,7,43]. But among diverse robotic flyers, few studies have implemented wing folding [10,11,21,44]. Of these, Wissa *et al*. [11] employed passive mechanisms, while Send *et al*. [10] implemented active wing folding with fixed amplitude and timing. Chen *et al*. [21] used a string-based approach to actuate variable-amplitude wing folding and found that wing folding helps reduce negative lift during upstroke in hovering conditions. Through PIV measurements along the streamwise centre plane of their robots, they demonstrated that the folded wings in upstroke are free of the impact of the low-pressure region under the wings, which otherwise would contribute to more negative lift.

Without wing folding ($\theta_{o}=0$), the positive lift generated during the downstroke is balanced by the negative lift produced during the upstroke (figure 5A). For a given Strouhal number, St, as wing folding, $\theta_{o}$, increases, we observe two benefits. First, handwing effective velocity increases because of the additional rotation with respect to the armwing and this enhanced velocity increases the local effective angle of attack. As a result, both factors can contribute to greater lift (snapshot ii, figure 3A(4) and figure 4). Second, the effective wing surface area is reduced during the upstroke, which alleviates negative lift, as has also been demonstrated in CFD simulations [45]. The additive effect of these factors results in a net positive cycle-averaged lift, $C_{L}$, that increases with the folding amplitude, $\theta_{o}$ (figure 5A).

We observe a qualitatively distinct phenomenon when wing folding amplitude $\theta_{o}$ exceeds a certain threshold (see schematics in figure 3C). The handwing lags the armwing and when the armwing transitions from down- to up-stroke, the handwing continues through its ‘downstroke’ trajectory. The two wingtips move towards one another as they approach the midline in a manner that can be described heuristically as ‘scooping’ the air, producing a trough of negative lift (figure 3A(4), between snapshots ii and iii). This phenomenon is also observed in small hovering birds [19,20].

As the handwings approach and ultimately clap (figure 3B), the air is propelled dorsally and ventrally from the small gap between the wings (figure 3C). The net effect is a downward, ventral, momentum jet that produces a positive lift, comparable in magnitude to that generated during downstroke (figure 3A(4)). In contrast, when Gouldian finches clap, they position their wings to prevent dorsally directed air movement [20]). One might question if the cycle-averaged lift improvement is not due to the ventral wing clapping but solely due to the change in wing kinematics. To resolve this question, we compared, for the case of St=0.41, three folding cases: $\theta_{o}$ = 0, 60 and 100, the lift and power measured experimentally with that predicted using the quasi-steady model with the same (measured) wing kinematics. While the quasi-steady model does capture the aerodynamic effects of the wing, it does not model wing–wing interactions and thus cannot predict the observed clapping phenomenon (figure 3B). This comparison confirms that the wing–wing interaction is responsible for the lift enhancement. As seen in supplementary material figure S5, when the handwings are widely apart on either side of the body and do not come close together, the predicted $C_{L}$ agrees very well with the measured values. However, when $\theta_{o}$= 100, the wing–wing interaction becomes important and the predicted $C_{L}$ is substantially lower than the measured value. On the other hand, for all cases, the predictions of the power coefficient, $C_{P}$, by the quasi-steady model closely match the measured values, indicating that the wing–wing interaction does not significantly contribute to the additional power expenditure.

The wingtips separate after the clap and are repositioned prior to the next downstroke (snapshot iv, figure 3A(4)). With no modification of the movement, this preparatory movement could incur a substantial negative lift penalty; however, birds and bats adopt similar mitigation strategies. In both groups, the handwing supinates (rotates about the spanwise long axis in the palm-upward direction) at the wrist and the wing retracts along the span by flexion at the elbow. The posture of the handwing may come close to a vertical plane before moving dorsally—as it ‘slices’ through the air—during the upstroke [2,4,8,46]. Upstroke kinematics of bats and birds reduce negative lift [2,19,39] and generate a small amount of thrust [36,47]. The wings of Flapperoo presented in this article can neither twist nor retract and the resulting unfavourable orientation of wing surfaces during rapid upstrokes leads to a substantial negative lift. However, we then designed, built and tested a Flapperoo with twisting capability and found that twisting significantly decreased both power consumption and negative lift during upstroke [48].

In summary, two different mechanisms to obtain optimal efficiency, characterized by power economy, are displayed here and we may classify them as symmetry-breaking lift augmentation (small folding amplitude $\theta_{o}$) and appendage-based jet propulsion (extreme folding amplitude $\theta_{o}$). At low speed or high St (figure 5B), it takes extra effort to actuate large wing folding to effect air jet for extra lift; thus, the symmetry-breaking strategy (moderate folding amplitude $\theta_{o}∼30^{\circ }$) is more cost-effective, in terms of $C_{L}/C_{P}$; on the other hand, for fast flight speed or low St, while the symmetry-breaking strategy is still rewarding, jet propulsion with a large folding angle becomes much more efficient (figure 6). It is remarkable to note that flapping wing flight has both strategies at its disposal.

### 4.4. Similarities and differences with ‘clap-and-fling’

The characteristic ‘clap-and-fling’ mechanism of insects and slowly flying birds [37,49,50] relies on the two wings coming together during the wingbeat cycle, but it differs fundamentally from the ventral clapping motion considered here. In clap-and-fling, the ‘fling’ part of the motion generates thrust and enhances lift by augmenting circulation around the wing during the early downstroke on the dorsal side [37]. The ventral clapping mechanism that we describe here generates lift during the upstroke by producing a directed jet of air.

In both clap-and-fling and ventral wing clapping, however, the negative lift is produced as a result of an upward jet, which can persist before and during the clapping ($C_{L}$ of case $\theta_{o}=100$, figure 3A(4),B) [20,37]. The magnitude of net lift increase for insects, due to clap-and-fling, is small (figure 3 in [37]), whereas we observe a more substantial boost, with a peak in the clapping-derived lift force comparable to that generated during the downstroke. This distinction might be due to differences in the swept area. In clap-and-fling, the wings simultaneously rotate and translate (panel M-P in fig. 7 in [37]) so that the leading edge of the wings meets first, subsequently ‘squeezing’ air out at the trailing edge. However, the volume of air trapped during this motion is much less than that of the corresponding volume of air trapped by Flapperoo, where the armwings never come close and the handwings are on opposite sides of the relatively large body.

### 4.5. Guidelines for flapping wing robots

The potential benefits of ventral wing clapping, as demonstrated here, may inform future designs of flapping-wing robots with high payload or endurance requirements. Wing folding in birds and bats is implemented primarily by flexion and extension at the wrist joint [51,52].

Even though a passive wrist joint with well-tuned stiffness to a specific speed may achieve beneficial folding angles, the flight efficiency may not adapt to the speed changes. For example, the cycle-averaged power consumption of one passively deforming, bird-inspired ornithopter, Park Hawk [53], was approximately 60 W, which is considerably higher than the 20 W power requirement reported for SmartBird, a seagull-inspired robot that performs active wing folding and twist [10,11], even though SmartBird has a greater wingspan and weighs more than Park Hawk. Here, we find that the cycle-averaged lift increases monotonically with the wing folding angle across St= 0.2–0.4 (figure 5A). This translates directly to the capacity to carry larger payloads and suggests that the energetic penalty for actuating the wing folding motion provides an effective compromise between generating lift and energetic cost (figure 5B).

The controlled, selective variation in wing folding angles, with flight speed by birds and bats [3,5,43], suggests that a flexible capability to modulate lift generation relative to energetic cost confers performance benefits. Adjustable folding angles could be incorporated in future designs for flapping wing robots to enhance their performance. For example, when agility is important, such as when navigating through a tight turn, an instantaneous boost of lift conferred by wing clapping might provide additional benefits with little impact on overall energetics due to the brief and temporary increase in power consumption. Moreover, ventral clapping is observed during fast flight (U=7.4 m s^−1^ or St∼0.2 [5]) in *Cynopterus brachyotis*, where a larger thrust is probably required to overcome high drag.

Indeed, the next generation of Flapperoo is also able to perform wing twists [48] and it demonstrates the air jet due to ventral wing clapping, as discussed in this article, which can be directed by wing angling at the moment of clap, where the leading edge almost touches but the trailing edge is positioned slightly apart. This angled clapping produces streamwise propulsion (thrust) in addition to the lift. This novel appendage-based propulsion offers many great opportunities to further probe into the optimal wing kinematics involving highly unsteady vortex dynamics and also offers new control authority in both lift and thrust directions.

## Data Availability

Raw data can be accessed here: [54]. Supplementary material is available online [55].

## Footnotes

^1^
Note that in this study, we employ a specific definition of wing folding: the additional rotation of the outboard wing section (handwing) relative to the inboard wing section (armwing) in the stroke plane (figure 1B) [10,11]. This differs from the planar protraction/retraction of wings along the wingspan [13], which has also been referred to as wing folding [14–16].

## References

[1] Muijres FT, Johansson LC, Barfield R, Wolf M, Spedding GR, Hedenström A. 2008 Leading-edge vortex improves lift in slow-flying bats. Science **319**, 1250–1253. (10.1126/science.1153019) 18309085

[2] Hubel TY, Riskin DK, Swartz SM, Breuer KS. 2010 Wake structure and wing kinematics: the flight of the lesser dog-faced fruit bat, Cynopterus brachyotis. J. Exp. Biol. **213**, 3427–3440. (10.1242/jeb.043257) 20889823

[3] Hubel TY, Hristov NI, Swartz SM, Breuer KS. 2016 Wake structure and kinematics in two insectivorous bats. Phil. Trans. R. Soc. B **371**, 20150385. (10.1098/rstb.2015.0385) 27528775 PMC4992709

[4] Fan X, Breuer K. 2022 Low-order modeling of flapping flight with highly articulated, cambered, heavy wings. AIAA J. **60**, 10. (10.2514/1.j060661) PMC9490335 36128710

[5] Fan X, Swartz S, Breuer K. 2022 Power requirements for bat-inspired flapping flight with heavy, highly articulated and cambered wings. J. R. Soc. Interface **19**, 20220315. (10.1098/rsif.2022.0315) 36128710 PMC9490335

[6] Riskin DK, Willis DJ, Iriarte-Díaz J, Hedrick TL, Kostandov M, Chen J, Laidlaw DH, Breuer KS, Swartz SM. 2008 Quantifying the complexity of bat wing kinematics. J. Theor. Biol. **254**, 604–615. (10.1016/j.jtbi.2008.06.011) 18621062

[7] Riskin DK, Bergou A, Breuer KS, Swartz SM. 2012 Upstroke wing flexion and the inertial cost of bat flight. Proc. R. Soc. B **279**, 2945–2950. (10.1098/rspb.2012.0346) PMC3385481 22496186

[8] Sekhar S, Windes P, Fan X, Tafti DK. 2019 Canonical description of wing kinematics and dynamics for a straight flying insectivorous bat (Hipposideros pratti). PLoS One **14**, e0218672. (10.1371/journal.pone.0218672) 31237912 PMC6592571

[9] Fan X, Breuer K, Vejdani H. 2021 Wing fold and twist greatly improves flight efficiency for bat-scale flapping wing robots. In IEEE Int. Conf. on Intelligent Robots and Systems, Prague, Czech Republic. (10.1109/IROS51168.2021.9636735)

[10] Send W, Fischer M, Jebens K, Mugrauer R, Nagarathinam A, Scharstein F. 2012 Artificial hinged-wing bird with active torsion and partially linear kinematics. In 28th Congress of the International Council of the Aeronautical Sciences, pp. 23–28. Edinburgh, UK: Optimage Ltd. on behalf of the International Council of the Aeronautical Sciences.

[11] Wissa AA, Tummala Y, Hubbard Jr JE, Frecker MI. 2012 Passively morphing ornithopter wings constructed using a novel compliant spine: design and testing. Smart Mater. Struct. **21**, 094028. (10.1088/0964-1726/21/9/094028)

[12] Bie D, Li D, Xiang J, Li H, Kan Z, Sun Y. 2021 Design, aerodynamic analysis and test flight of a bat-inspired tailless flapping wing unmanned aerial vehicle. Aerosp. Sci. Technol. **112**, 106557. (10.1016/j.ast.2021.106557)

[13] Vejdani HR, Boerma DB, Swartz SM, Breuer KS. 2019 The dynamics of hovering flight in hummingbirds, insects and bats with implications for aerial robotics. Bioinspir. Biomim. **14**, 016003. (10.1088/1748-3190/aaea56) 30411710

[14] Bahlman JW, Swartz SM, Breuer KS. 2014 How wing kinematics affect power requirements and aerodynamic force production in a robotic bat wing. Bioinspir. Biomim. **9**, 025008. (10.1088/1748-3182/9/2/025008) 24851830

[15] Bahlman JW, Swartz SM, Breuer KS. 2013 Design and characterization of a multi-articulated robotic bat wing. Bioinspir. Biomim. **8**, 016009. (10.1088/1748-3182/8/1/016009) 23385471

[16] Stowers AK, Lentink D. 2015 Folding in and out: passive morphing in flapping wings. Bioinspir. Biomim. **10**, 025001. (10.1088/1748-3190/10/2/025001) 25807583

[17] Gould E. 1988 Wing-clapping sounds of Eonycteris spelaea (Pteropodidae) in Malaysia. J. Mammal. **69**, 378–379. (10.2307/1381392)

[18] Boonman A, Bumrungsri S, Yovel Y. 2014 Nonecholocating fruit bats produce biosonar clicks with their wings. Curr. Biol. **24**, 2962–2967. (10.1016/j.cub.2014.10.077) 25484290

[19] Chang YH, Ting SC, Liu CC, Yang JT, Soong CY. 2011 An unconventional mechanism of lift production during the downstroke in a hovering bird (Zosterops japonicus). Exp. Fluids **51**, 1231–1243. (10.1007/s00348-011-1145-8)

[20] Chang YH, Ting SC, Su JY, Soong CY, Yang JT. 2013 Ventral-clap modes of hovering passerines. Phys. Rev. E **87**, 022707 1–11. . (10.1103/physreve.87.022707) 23496548

[21] Chen WH, I.Yeh S. 2021 Aerodynamic effects on an emulated hovering passerine with different wing-folding amplitudes. Bioinspiration Biomimetics **16**. (10.1088/1748-3190/abf6b8) 33836515

[22] Kashi E, A.Kulkarni A, Perrotta G, C.Leftwich M. 2020 Flowfields produced by a robotic sea lion foreflipper starting from rest. Bioinspiration Biomimetics **15**. (10.1088/1748-3190/ab6a62) 31923905

[23] Vaughan TA. 1959 Functional morphology of three bats: Eumops, Myotis and Macrotus. Univ. Kans. Sci. Bull. **12/1**, 1–153.

[24] Taylor GK, Thomas ALR. 2003 Dynamic flight stability in the desert locust Schistocerca gregaria. J. Exp. Biol. **206**, 2803–2829. (10.1242/jeb.00501) 12847126

[25] Taylor GK, Nudds RL, Thomas ALR. 2003 Flying and swimming animals cruise at a Strouhal number tuned for high power efficiency. Nature **425**, 707–711. (10.1038/nature02000) 14562101

[26] Bohl DG, Koochesfahani MM. 2009 MTV measurements of the vortical field in the wake of an airfoil oscillating at high reduced frequency. J. Fluid Mech. **620**, 63–88. (10.1017/s0022112008004734)

[27] Mathai V, Das A, Naylor DL, Breuer KS. 2023 Shape-morphing dynamics of soft compliant membranes for drag and turbulence modulation. Phys. Rev. Lett. **131**, 114003. (10.1103/physrevlett.131.114003) 37774286

[28] Breuer KS, Drela M, Fan X, di Luca M. 2022 Design and performance of an ultra-compact low-speed low-turbulence level wind tunnel for aerodynamic and animal flight experiments. Exp. Fluids **63**, 169. (10.1007/s00348-022-03519-1)

[29] Hedrick TL. 2008 Software techniques for two- and three-dimensional kinematic measurements of biological and biomimetic systems. Bioinspir. Biomim. **3**, 034001. (10.1088/1748-3182/3/3/034001) 18591738

[30] Onoue K, Breuer KS. 2016 Vortex formation and shedding from a cyber-physical pitching plate. J. Fluid Mech. **793**, 229–247. (10.1017/jfm.2016.134)

[31] Savitzky Abraham, Golay MJE. 1964 Smoothing and differentiation of data by simplified least squares procedures. Anal. Chem. **36**, 1627–1639. (10.1021/ac60214a047) 22324618

[32] Costello JH, Colin SP, Dabiri JO, Gemmell BJ, Lucas KN, Sutherland KR. 2021 The hydrodynamics of jellyfish swimming. Annu. Rev. Mar. Sci. **13**, 375–396. (10.1146/annurev-marine-031120-091442) 32600216

[33] Anderson EJ, DeMont ME. 2000 The mechanics of locomotion in the squid Loligo pealei: locomotory function and unsteady hydrodynamics of the jet and intramantle pressure. J. Exp. Biol. **203**, 2851–2863. (10.1242/jeb.203.18.2851) 10952883

[34] Perrotta G, Fish FE, Adams DS, Leahy AM, Downs AM, Leftwich MC. 2021 Velocity field measurements of the California sea lion propulsive stroke using bubble PIV. Fluids **7**, 3. (10.3390/fluids7010003)

[35] Shyy W, Aono H, Kang CK, Liu H. 2013 An introduction to flapping wing aerodynamics. Cambridge, UK: Cambridge University Press.

[36] Hedenström A, Johansson LC, Wolf M, von Busse R, Winter Y, Spedding GR. 2007 Bat flight generates complex aerodynamic tracks. Science **316**, 894–897. (10.1126/science.1142281) 17495171

[37] Lehmann FO, Sane SP, Dickinson M. 2005 The aerodynamic effects of wing–wing interaction in flapping insect wings. J. Exp. Biol. **208**, 3075–3092. (10.1242/jeb.01744) 16081606

[38] Sane SP, Dickinson MH. 2002 The aerodynamic effects of wing rotation and a revised quasi-steady model of flapping flight. J. Exp. Biol. **205**, 1087–1096. (10.1242/jeb.205.8.1087) 11919268

[39] Windes P, Fan X, Bender M, Tafti DK, Müller R. 2018 A computational investigation of lift generation and power expenditure of Pratt’s roundleaf bat (Hipposideros pratti) in forward flight. PLoS One **13**, e0207613. (10.1371/journal.pone.0207613) 30485321 PMC6261594

[40] Fan X, Breuer K. 2021 Reduced-order modeling of a bat flying with heavy and highly articulated flapping wing. In AIAA Scitech 2021 Forum, virtual event, pp. 1–14. Reston, VA. (10.2514/6.2021-0344)

[41] Dickinson MH, Lehmann FO, Sane SP. 1999 Wing rotation and the aerodynamic basis of insect flight. Science **284**, 1954–1960. (10.1126/science.284.5422.1954) 10373107

[42] Wang ZJ. 2000 Vortex shedding and frequency selection in flapping flight. J. Fluid Mech. **410**, 323–341. (10.1017/s0022112099008071)

[43] Parslew B. 2012 Simulating avian wingbeats and wakes. J. Biomech. **45**, S7. (10.1016/S0021-9290(12)70008-8) 20732684

[44] Qin S, Weng Z, Li Z, Xiang Y, Liu H. 2021 On the controlled evolution for wingtip vortices of a flapping wing model at bird scale. Aerosp. Sci. Technol. **110**, 106460. (10.1016/j.ast.2020.106460)

[45] Lang X, Song B, Yang W, Yang X. 2022 Effect of spanwise folding on the aerodynamic performance of three dimensional flapping flat wing. Phys. Fluids **34**. (10.1063/5.0078844)

[46] Hedrick TL, Usherwood JR, Biewener AA. 2004 Wing inertia and whole-body acceleration: an analysis of instantaneous aerodynamic force production in cockatiels (Nymphicus hollandicus) flying across a range of speeds. J. Exp. Biol. **207**, 1689–1702. (10.1242/jeb.00933) 15073202

[47] Muijres FT, Christoffer Johansson L, Winter Y, Hedenström A. 2014 Leading edge vortices in lesser long-nosed bats occurring at slow but not fast flight speeds. Bioinspir. Biomim. **9**, 025006. (10.1088/1748-3182/9/2/025006) 24855067

[48] Fan X, Gehrke A, Breuer K. 2024 Wing twist and folding work in synergy to propel flapping wing animals and robots. In IEEE/RSJ Int. Conf. on Intelligent Robots and Systems. (10.1109/IROS58592.2024.10801820)

[49] Weis-Fogh T. 1973 Quick estimates of flight fitness in hovering animals, including novel mechanisms for lift production. J. Exp. Biol. **59**, 169–230. (10.1242/jeb.59.1.169)

[50] Crandell KE, Tobalske BW. 2015 Kinematics and aerodynamics of avian upstrokes during slow flight. J. Exp. Biol. **218**, 16. (10.1242/jeb.116228) 26089528

[51] Pennycuick. 2008 Modelling the flying bird. Burlington MA: Academic Press. (10.1016/S1875-306X(08)00001-4)

[52] Norberg UML. 1990 Vertebrate flight: mechanics, physiology, morphology, ecology and evolution. Zoophysiology. vol. 27. Berlin, Germany: Springer-Verlag.

[53] Kinkade AS. Ornithopter. Patent US20020173217A1, November 2002. Patent Application. https://patents.google.com/patent/US20020173217A1

[54] Fan X. 2025 Raw data [Data set]. Zenodo (10.5281/zenodo.14598614)

[55] Fan X, Bortoni A, Hao S, Swartz SM, Breuer KS. 2025 Supplementary material from: Upstroke wing clapping in bats and bat-inspired robots offers efficient lift generation. Figshare. (10.6084/m9.figshare.c.7630665) PMC11837331 39968874

